# The multifaceted role of CARD9 in inflammatory bowel disease

**DOI:** 10.1111/jcmm.14770

**Published:** 2019-11-06

**Authors:** Ping Luo, Zhiwen Yang, Bin Chen, Xiaoming Zhong

**Affiliations:** ^1^ Department of Breast Surgery Nanchang Third Hospital Nanchang China; ^2^ Department of Pharmacy Songjiang Hospital Affiliated Shanghai First People’s Hospital Shanghai Jiao Tong University Shanghai China; ^3^ Surgery Department First Affiliated Hospital of Gannan Medical University Ganzhou China; ^4^ Jiangxi Province Tumor Hospital Nanchang China

**Keywords:** CARD9, inflammatory bowel disease, predisposing and protective variants, therapy

## Abstract

Inflammatory bowel disease (IBD) involves a dysregulated immune response to the gut microbiota. Emerging evidence has demonstrated that dysfunctions in caspase recruitment domain‐containing protein 9 (CARD9) may contribute to the pathogenesis of IBD. Interestingly, an allelic series of *Card9* variants have both a common predisposing and rare protective function in IBD patients. In this review, we provide mechanistic insights into the role of the CARD9 adaptor molecule in intestinal inflammation and determine a potential CARD9‐targeting therapeutic approach against IBD.

## 1 INTRODUCTION

**Figure 1:**
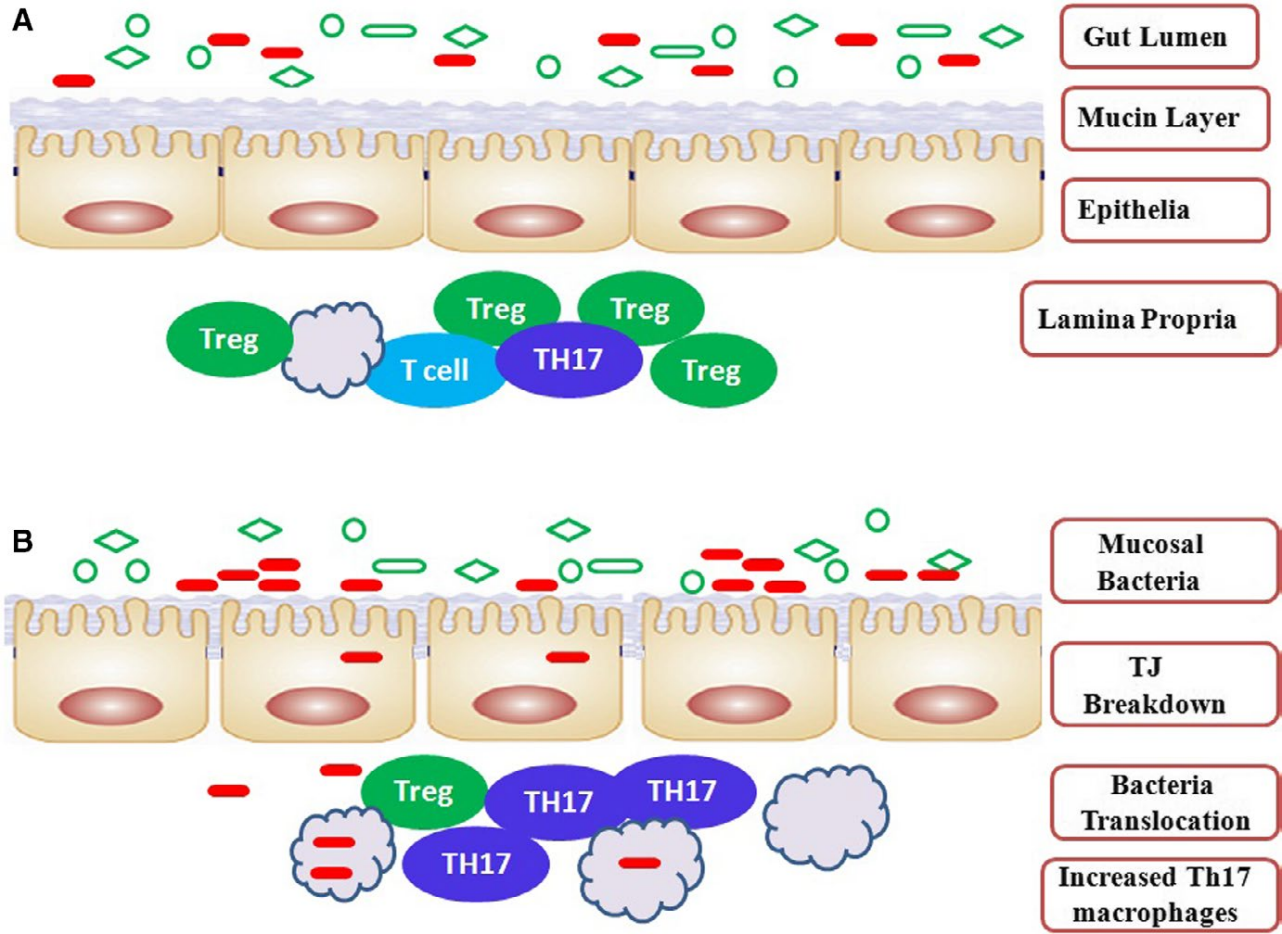
Topology of inflammation in IBD. A, Health status. The intestinal immune system protects the largest mucosal surface against infection and injury; B, IBDs. Abnormal intestinal immunity is thought to lead to the development of IBD

Inflammatory bowel diseases (IBDs) including Crohn's disease (CD) and ulcerative colitis (UC) are chronic immune‐mediated inflammatory diseases of the intestinal tract. Although the exact aetiology of IBD remains unclear, it is thought to involve a close relationship between immunity, genetic and environmental factors. It is well‐known that intestinal commensal microorganisms maintain homeostasis under the surveillance of the host immune system. A compromise in the integrity of the intestinal epithelial barrier results in detrimental microbial invasion and disrupts the beneficial host‐microbe balance in the intestinal tract. This deficiency facilitates detrimental microbial invasion and disrupts the beneficial host‐microbe balance in the intestinal tract. Intestinal mucosal immune cells eventually induce an aberrant immune response against the gut microbiota, leading to a higher risk of developing IBD^1^ (Figure 1).

Innate immune cells recognize evolutionarily conserved microbial molecules through pattern recognition receptors (PRRs), such as C‐type lectin receptors (CLRs) and Toll‐like receptors (TLRs). These PRRs on the surface of innate cells effectively sense distinct microbial components and can trigger an inflammatory cascade in a CARD9‐dependent manner. CARD9 is required for TLR pathways to activate mitogen‐activated protein kinase (MAPK) and CLRs pathways to activate nuclear factor‐kappaB (NF‐κB). As a result, CARD9, a signalling adaptor known to regulate innate immune activation, plays a major role in the sensing of pathogenic microorganisms.^2^

Emerging evidence has demonstrated that CARD9 dysfunction may contribute to the pathogenesis of IBD.^3^, ^4^ Unlike other IBD risk genes, *Card9* alleles in IBD patients have both a common predisposing and rare protective function. In this review, we summarize recent progress on the molecular mechanisms of CARD9 in IBD and discuss the feasibility of small‐molecule inhibitors targeting CARD9 for the treatment of IBD.

## 2 THE DUAL ROLE OF *CARD9* GENETIC MUTATIONS IN IBD PATIENTS

In 2008, a functional candidate gene analysis of the innate immune pathway was used to investigate *Card9* rs10870077, the intronic substitution of base C (cytosine) for G (guanine) in the *Card9* genetic locus on chromosome 9. The variant rs10870077 in the *Card9* locus was found to be a high‐risk factor for phase III IBD patients, showing a strong association with UC and a moderate association with CD.^5^ This was the first time that the *Card9* gene was identified as a risk factor for IBD patients. In 2012, genome‐wide association studies were conducted to analyse *Card9* single‐nucleotide polymorphisms from IBD patients. In this study, *Card9* variant rs4077515 known to create substitution S12N was established as a risk factor in the development of ileal CD, which initiated an innate immune response to peptidoglycan, a bacterial cell wall macromolecule.^6^ In 2018, another variant *Card9* rs10781499 was confirmed to be a high IBD genetic risk factor, which altered the composition of the gut microbiota in patients with IBD.^7^

In contrast to the above mentioned studies, some rare variants of *Card9* have also been identified as protective against IBD. A rare splicesite variant, c.IVS11 + 1G>C with the substitution of G for C at position 1 of exon 11 of the *Card9* gene, suggested a potentially strong protective effect against disease development. Of note, c.IVS11 + 1G>C (rs141992399) as a protective variant actually occurs on a haplotype carrying the risk allele at S12N, indicating that not only were the two associations independent, but that the splice variant also completely eliminated the risk normally associated with the common haplotype. As the CD risk allele at S12N may lead to higher expression of CARD9, a consistent allelic series existed if the splice variant was substantially low or non‐functional and therefore highly protective.^8^, ^9^ Similarly, a rare nonsynonymous variant, *Card9* rs200735402 with the substitution of C for T at position 139265120, was also shown to have a functionally protective role in CD patients.^10^

**Figure 2:**
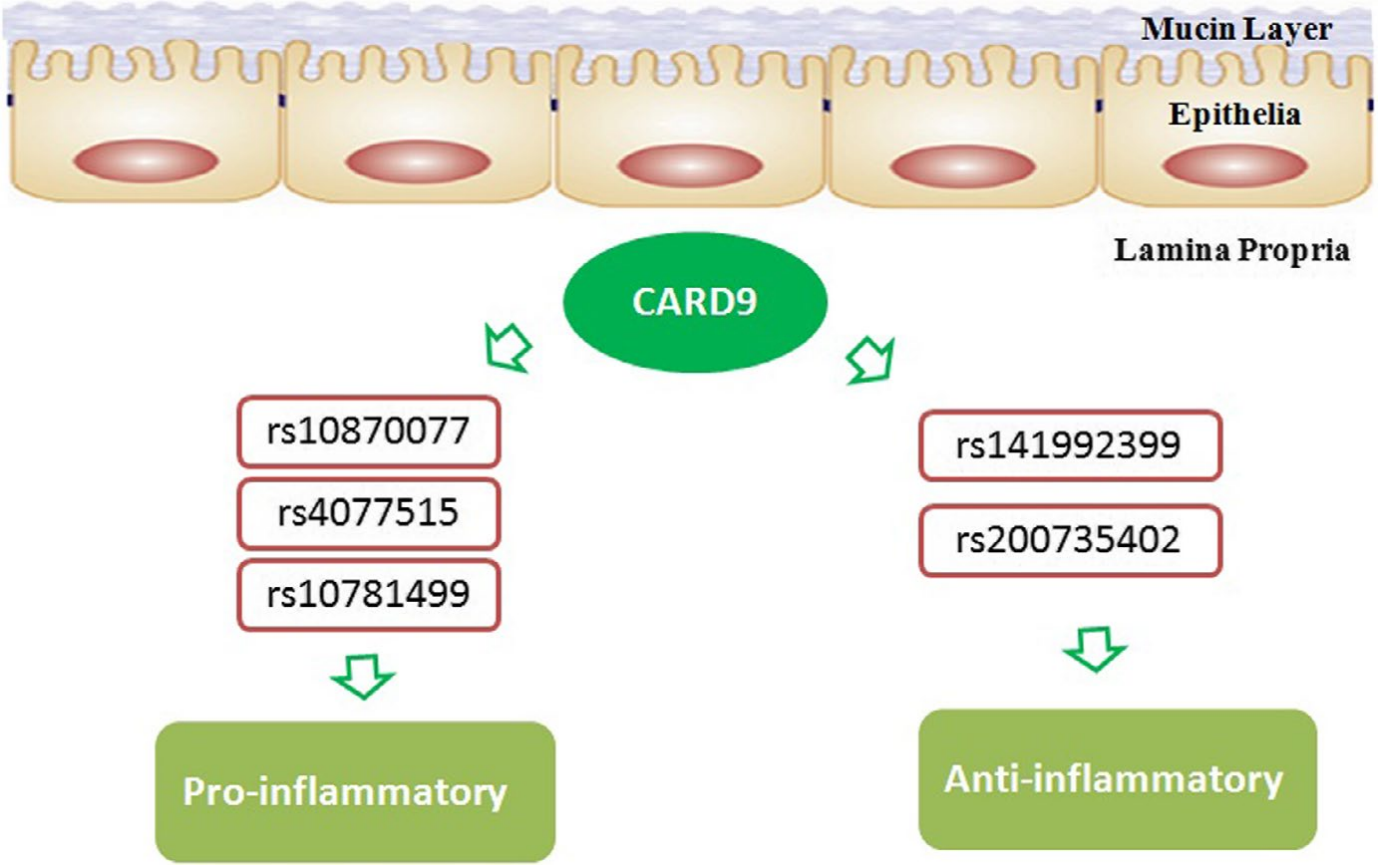
The dual role of *Card9* genetic mutations in IBD patients. *Card9* alleles in IBD patients have both a common predisposing and rare protective function. The rs10870077, rs4077515 and rs10781499 variants in *Card9* were confirmed to be high genetic risk factors, but rs141992399 and rs200735402 were shown to have a functional protective role. Of note, c.IVS11 + 1G>C (rs141992399) is a protective variant and occurs on a haplotype carrying the risk allele at S12N

Some *Card9* variants exhibited an increased risk while others were shown to have a protective effect on IBD (Figure 2). This can be attributed to the *Card9* variants having different mechanisms of pathogenesis, and thus different disease susceptibilities. CARD9 contains a protein interaction domain known to participate in the activation or suppression of other CARD‐containing proteins.^10^ The *Card9* gene variant is likely to change the protein function or structure level in a positive and negative manner. Therefore, the predisposing variants (rs10870077, rs10781499 and rs4077515) were associated with increased expression of CARD9 mRNA and activation of the immune response to pathogens.^5^, ^11^ The protective variants, c.IVS11 + 1G>C and rs200735402, may have lost the biological function of CARD9, inhibiting the immune response to the pathogens.^5^, ^11^

Different from the above study, a recent study reported that *Card9* predisposing variants, rs10870077 and rs10781499, did not increase susceptibility to IBD in the Chinese Han population. These discrepancies may be partly explained by the different incidences, epidemiologies and phenotypes among patients from the Chinese Han population and Western countries.^12^

## 3 CARD9 DEFICIENCY IMPAIRED IMMUNE RESPONSES

Dextran sulphate sodium (DSS) is often used to induce an UC model in mice, which exhibits several clinical and histological features similar to human UC.^13^ Following DSS administration, *Card9*‐null mice had an impaired immune response, defective expression of interleukin (IL)‐6, IL‐17A, IL‐22 and regenerating islet‐derived 3 gamma (RegIIIγ).^3^ IL‐6 protected against intestinal epithelium injury by secreting intestinal trefoil factor and antimicrobial peptides.^14^, ^15^ IL‐17A increased the expression of colonic β‐defensin.^16^ IL‐22‐mediated STAT3 signalling was involved in mucosal wound healing of intestinal epithelial cells.^17^ RegIIIγ in epithelial cells acts as an antibacterial protein facilitating intestinal epithelial wound healing by preventing wound infection and inflammation.^18^ In addition, *Card9*‐null mice were also found to have fewer colonic T‐helper (Th) 17 cells and exhibited impaired Th17 responses. As predicted, DSS‐induced colitis in *Card9* ^−/−‐^ mice inhibited intestinal epithelial restoration and impaired gut recovery by significantly increasing apoptosis and reducing proliferation of intestinal epithelial cells.^4^ Given the impaired immune responses and injured intestinal epithelial cells, *Card9*‐null mice were less efficient in controlling fungal colonization, increasing their susceptibility to intestinal inflammation.^3^, ^4^

**Figure 3:**
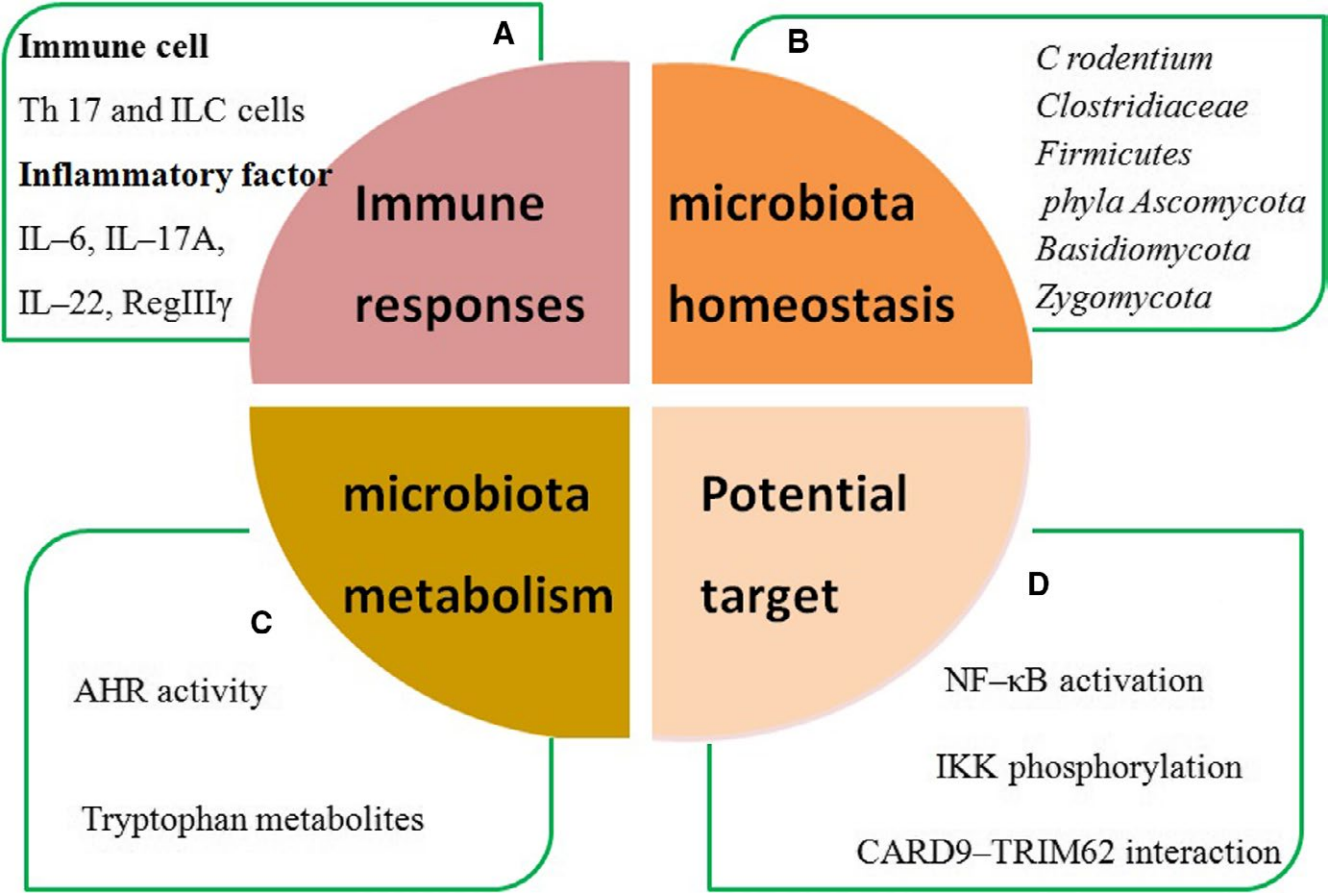
The potential functions of *Card9* in IBD. A, CARD9 deficiency impaired immune responses; B, CARD9 controlled gut microbiota; C, CARD9 altered gut microbiota metabolism; D, the positive therapeutic effect of CARD9‐targeting small‐molecule inhibitor

*Citrobacter rodentium* infection in mice can induce acute, self‐limited colitis. *C rodentium* is a Gram‐negative and murine‐specific bacterial pathogen that is equivalent to the human enteropathogenic *Escherichia coli*.^19^ During *C rodentium* infection, *Card9*‐null mice were found to have an impaired immune response along with defective expression of colonic IL‐6, IL‐17A, IL‐22 and RegIIIγ. To further investigate the gut immune response elicited by *C rodentium*, antigen‐specific T cell cytokine production in mesenteric lymph nodes (MLNs) was assessed, which suggested that the inappropriate or ongoing inflammation in *Card9* knockout mice was characterized by decreased levels of IFNγ and Th17 cytokine responses. As reported previously, innate lymphoid cells (ILCs) in the gut also secrete IL‐17A and IL‐22 cytokines, particularly in response to *C rodentium* infection.^20^, ^21^ The Th17‐dependent *C rodentium* colitis model showed that Card9 deletion leads to significantly fewer CD3^+^CD4^+^IL^−^17A^+^ Th17 cells and CD3^−^CD4^−^NKP46^+^ ILC populations. Card9 is thus required for both Th17 cells and ILC‐mediated intestinal immune responses. As a result, *Card9*‐null mice were unable to control bacterial propagation and were more susceptible to intestinal inflammation during *C rodentium* infection (Figure 3A).^3^

## 4 CARD9 CONTROLLED GUT MICROBIOTA

*Card9,* as an IBD susceptibility gene, is required to shape the normal gut microbiota.^3^ However, it also raises a question about the specific role of the gut microbiota in susceptibility to colitis. To address this question, *C rodentium*‐induced and DSS‐induced colitis mouse models were used in the following studies (Figure 3B).

In *C rodentium*‐induced colitis (Table 1), CARD9 effectively controlled the virulence of *C rodentium* to promote pathogen eradication through both gut microbiota‐independent and microbiota‐dependent mechanisms.^22^ Previous studies reported that the specific intestinal IgG response was required to eliminate *C rodentium* infection.^23^ However, *Card9* ^−/−^ (*Card9* ^−/−^→germ free) mice in this study failed to stimulate the *C rodentium*‐specific IgG response. As a result, *Card9* ^−/−^ mice were associated with a defective intestinal humoral immune response and exhibited a higher susceptibility to *C rodentium* in the late‐phase of infection. This finding indicated that CARD9 controlled pathogen virulence by supporting a specific humoral response in a microbiota‐independent manner. Several studies have shown that the gut microbiota also played a key role in the clearance of *C rodentium.* 24, 25 Resistance to *C rodentium* infection was associated with a decrease in Firmicutes,^26^ and an increase in Clostridiaceae.^27^ Interestingly, CARD9 in *C rodentium*‐induced colitis mice decreased the levels of Clostridiaceae and increased the levels of Firmicutes in the baseline faecal bacterial composition. This result revealed a defective ecological effect in shaping a balanced intestinal microbiota in *Card9* ^−/−^ mice. Furthermore, CARD9 was required to shape the intestinal microbiota that competed for sugars with *C rodentium*. A polysaccharides‐enriched diet could override the susceptibility of *Card9* ^−/−^ mice to intestinal *C rodentium* colonization and alleviate infection severity. Diet intervention in *Card9* ^−/−^ mice was attributed to shaping a favourable gut microbiota and promoting a *C rodentium*‐specific antibody response. Thereby, CARD9 modulated the susceptibility to intestinal *C rodentium* infection in a microbiota‐dependent manner.

**Table 1:** The role of *Card9* in *Citrobacter rodentium* and DSS‐induced colitis mouse models

Mice model	Type of model	Gene	Key cytokines	Immune cells	Intestinal epithelial cells	Gut homeostasis	Gut microbiota metabolism	References
DSS Colitis	Chemical	*card9*	IL–6					
			IL–17A	Th17	Impairment	Fungal microbiota	Tryptophan AHR ligands	11,17
			IL–22					
			RegIIIγ					
*C rodentium*	Infectious	*card9*	IL–6					
			IL–17A	Th17	–	Microbiota–independent and dependent mechanism	–	11,21
			IL–22	ILCs				
			RegIIIγ					

Abbreviations: AHR, aryl hydrocarbon receptor; ILCs, innate lymphoid cells; Th17, T‐helper cell 17.

In DDS‐induced colitis (Table 1), CARD9 also had a key role in shaping the ecosystem of bacteria and fungi in the gut. The faecal fungal composition in *Card9* ^−/−^ mice was changed and was dominated by members of the phyla *Ascomycota*, *Basidiomycota* and *Zygomycota*. In parallel, composition of the faecal bacterial microbiota in *Card9* ^−/−^ mice was altered, but the alterations were less than those for fungi. These findings indicate that the susceptibility gene *Card9* controls fungal microbiota expansion for gut homeostasis in DDS‐induced colitis.^4^

## 5 CARD9 ALTERED GUT MICROBIOTA METABOLISM

Tryptophan catabolites from gut microbiota play a key role in intestinal mucosal immune responses, in which they act as aryl hydrocarbon receptor (AHR) ligands by modulating the production of IL‐22.^28^ In this study, the gut bacteria of *Card9* ^−/−^ mice failed to metabolize tryptophan into AHR ligands, leading to defective AHR activation and aggravation of intestinal inflammation. Administration of an AHR agonist can rescue the susceptibility of *Card9* ^−/−^→germ free mice to colitis. Moreover, AHR ligand production was normalization in the presence of exogenous IL‐22, which effectively relieved colitis susceptibility in *Card9* ^−/−^ mice. Following analysis of faecal samples, reduced AHR activity and tryptophan metabolites were observed in IBD patients, particularly in those with *Card9* rs10781499 as a risk allele associated with IBD. Consequently, these results provided strong evidence that CARD9 was required for intestinal microbiota metabolism and production of AHR agonists, altering the gut immune response that resulted in the loss of intestinal homeostasis (Figure 3C).^4^

## 6 CARD9 ACTIVATION WAS REQUIRED FOR TRIM62‐MEDIATED UBIQUITINATION

CARD9 is structurally similar to the CARMA family members, but lacks the C‐terminal MAGUK motif, and contains an amino‐terminal CARD and a carboxy‐terminal coiled‐coil domain.^29^ The N‐terminal CARD region of CARD9, comprising 7–98 amino acids, is involved in the association of CARD motifs, with an increased susceptibility to microbial infection.^2^, ^30^ However, the biological functions of the C‐terminus of CARD9 are unknown.

CARD9 S12NΔ11, located in the C‐terminal portion of the protein, was defined as a rare splice variant in which exon 11 of CARD9 is deleted. It provided strong protection against IBD in two previous studies.^8^, ^9^ In order to determine its potential mechanism, CARD9 S12NΔ11, as a protective variant, was used to investigate the biological functions of CARD9 in IBD. Interestingly, CARD9 S12NΔ11 was responsible for a dominant negative effect on CARD9 function, exhibiting impaired CARD9‐mediated cytokine production via direct hetero‐oligomerization interacting with full‐length CARD9. As CARD9 is a scaffold protein and coordinates the assembly of a multiprotein complex, it was determined whether loss of specific protein‐protein interactions by the CARD9 C‐terminal portion might alter its function. It should be noted that TRIM62 is a novel CARD9 interactor and can directly bind the C‐terminus of CARD9. Furthermore, the presence of CARD9 S12NΔ11 could effectively disrupt the CARD9‐TRIM62 interaction. As a result, CARD9‐dependent signalling pathways were blocked. TRIM62 generally modified their targeted proteins via several types of ubiquitin linkages, mainly K48‐ and K63‐based linkages that contributed to degradation and stabilization of protein complexes.^31^ The findings suggest that TRIM62‐induced CARD9 ubiquitination is dependent on K27‐based linkages. Importantly, TRIM62 induced CARD9 ubiquitination at residue K125, and CARD9 mutation at K125R abrogated CARD9‐mediated cytokine production. Furthermore, 33 311 clinical cases with the protective Δ11 splice variant were less likely to develop IBD regardless of the presence of the predisposing S12N mutation, which further verified an important biological role for the C‐terminus of CARD9. Therefore, we report here, for the first time, that the C‐terminus of CARD9 is a key regulatory module for efficient control of CARD9 activity and TRIM62 is a novel interactor of the CARD9 C‐terminus. The CARD9 protective variant did not undergo ubiquitination by TRIM62, leading to the detection of specific CARD9‐TRIM62 interactions and strong protection against IBD.^11^

## 7 CARD9‐TARGETED THERAPY WITH SMALL‐MOLECULE INHIBITORS

Inflammatory bowel disease is a chronic inflammatory disorder with abnormal humoral immune responses to gut commensal bacteria. Due to limited therapeutic efficacy and severe side effects, biological agents and immune suppressants are not the best treatment options for controlling disease development.^32^ To date, guiding the discovery of targeted therapeutics for IBD remains a major challenge.

*Card9* variants were found to have significant associations with IBD. Due to a loss of the key C‐terminal functions, the rare protective variant CARD9Δ11 disrupted the CARD9‐TRIM62 interaction, and inhibited NF‐κB activation, eventually showing a strong protective effect against IBD.^11^ These findings provided crucial clues that small‐molecule inhibitors, targeting and disrupting the direct interaction between CARD9 and TRIM62, may mimic the CARD9Δ11 protective actions in IBD. To address the challenge of targeting and disrupting the CARD9‐TRIM62 protein‐protein interaction, a set of 132 813 small molecules were collected to investigate the specificity using a multiplexed high‐throughput screen. Interestingly, four structurally related compounds, BRD5529, BRD4203, BRD8991 and BRD4098, emerged as effective small‐molecule inhibitors, which selectively interfere with CARD9‐TRIM62 at the CARD9 C‐terminus, not the CARD9‐BCL10 interaction at the CARD9 N terminus. After directly binding to CARD9 protein, small‐molecule compounds mimic the behaviour of the CARD9Δ11 protective variant by inhibiting TRIM62‐mediated CARD9 ubiquitination. Finally, when innate immune cells (bone marrow derived‐dendritic cells and THP‐1 monocytes) were treated with BRD5529, IKK phosphorylation and NF‐κB activation were attenuated in a CARD9‐dependent manner, supporting the concept of the CARD9‐targeting molecule (Figure 3D).^33^

Patients with complete CARD9 functional deficiency via missense or early stop codon mutations are susceptible to life‐threatening microbial infections.^33^ In the current work, these lead compounds provide precision immunomodulatory intervention rather than broad immunosuppression. Thus, targeting CARD9 with small molecules that mimicked the protective CARD9Δ11 mechanism, compared favourably with complete ablation of CARD9 activity, and may be a safe and effective targeted treatment.

## 8 CONCLUSIONS

CARD9 is a central integrator in innate immune cell activation, which triggers the inflammatory signalling pathway in response to microbial infection. Along with an in‐depth understanding of pathological CARD9 signalling, CARD9 was confirmed to play an essential role in the pathogenesis of inflammatory bowel diseases. CARD9, as a susceptibility factor for colitis, mainly induces the intestinal mucosal immune response and mediates gut microbiota composition and metabolism. Interestingly, an allelic series of *Card9* variants have both a common predisposing and rare protective function in IBD patients. These rare protective variants may provide crucial clues in order to achieve targeted treatments and improve current clinical approaches. Some lead compounds were confirmed to mimic the protective CARD9Δ11 mechanism that lost the key C‐terminal functions. These findings provide a novel CARD9‐targeting approach towards improved IBD therapeutics.

However, some important questions in this field still remain. The gut commensal microbiota contains a complex population of microorganisms, and CARD9 deficiency in IBD patient results in their being highly predisposed to fungal infections. It is unclear how CARD9 can reestablish intestinal epithelial homeostasis and restore beneficial bacterial colonization after inflammatory injury. Considering the close connection between protective genetic variants and therapeutic advances, this connection may be used as a clinical guide for the rational design of IBD therapeutics. The present work also showed a positive therapeutic effect of CARD9‐targeting small‐molecule inhibitor in the in vitro cellular model. However, it is unknown whether these lead compounds could progress to clinical practice as further data on human and experimental colitis are required.

## CONFLICT OF INTEREST

The authors declare that they have no conflict of interest.

## AUTHOR CONTRIBUTION

Z‐X M and C‐B designed and wrote the manuscript. Y‐Z W collected literature. L‐P edited and prepared the manuscript for submission. All authors read and approved the final manuscript.
